# Interprofessional Team Performance in Pediatric Settings: Analyzing Reflections of Quality Improvement (QI)-Trained Medical Students

**DOI:** 10.7759/cureus.90801

**Published:** 2025-08-23

**Authors:** Catherine Napolitano, Russell Buzalko, Gary L Beck Dallaghan, Katherine Mason, Kari A Simonsen

**Affiliations:** 1 Medical Education, Brown University, Providence, USA; 2 Medical Education, Children's Hospital and Medical Center, Omaha, USA; 3 Dean's Office, Carle Illinois College of Medicine, Urbana, USA; 4 Pediatrics, Brown University/Hasbro Children's Hospital, Providence, USA; 5 Pediatrics, University of Nebraska Medical Center, Omaha, USA

**Keywords:** effective communication, interprofessional education (ipe), medical student clerkship, multi-disciplinary team, quality improvement tool, teamstepps

## Abstract

Background and objective

Navigating interprofessional team dynamics is essential for high-quality patient care in pediatric settings. This study involved medical students on a pediatric clerkship who explored the characteristics of high- and low-performing clinical teams by considering drivers and barriers to effective team performance. By analyzing these reflections, the study aimed to identify key facilitators and barriers to effective team-based care.

Methods

Survey evaluations and narrative reflections were completed by third-year students (M3s) at a single US allopathic medical school during their pediatric clerkship after receiving training in TeamSTEPPS® and Institute for Healthcare Improvement (IHI) Open School, two programs that support quality improvement (QI) in healthcare. Descriptive statistical and inductive thematic analyses were conducted on the resulting 183 narratives. A valence rating system was employed to quantify narrative responses as positive/attractive or negative/aversive, with a Cronbach alpha of 0.958 between two independent reviewers.

Results

Inductive thematic analysis generated 40 themes that we grouped under the five TeamSTEPPS® skill domains (situation monitoring, communication, leadership, team structure, mutual support) into thematic conceptual models. High-performing teams demonstrated open communication, role clarity, shared understanding, and organized task delegation. Low-performing teams displayed a lack of information exchange, uncertain team roles, unhealthy power dynamics, and disorganized task delegation.

Conclusions

After instruction in QI methods, pediatric clerkship students identified consistent drivers of and barriers to effective team performance. The themes within the narrative reflections can provide insights into improving patient care delivery, specifically around situation monitoring, communication, and team structure.

## Introduction

The medical profession is constantly evolving, and ensuring continuous quality improvement (QI) is a crucial part of modern healthcare [1]. A psychologically safe environment, which includes a culture supportive of all team members speaking up about quality concerns, is an important construct within healthcare QI methodology and medical student education [2-6]. Interprofessional staff perspectives have previously been explored through the lens of participants speaking up in a clinical environment [5-8]. We expanded upon existing studies by focusing on medical students' observations of interprofessional teamwork and communication after providing dedicated instruction on these topics to better understand medical students’ views of team performance as contributors to patient care. A majority of interprofessional education sessions occur in pre-clinical medical student education [9]; therefore, clinical medical student insights can help drive improvements in patient care delivery and experiential learning during academic team-based rounds.

TeamSTEPPS® 2.0 and Institute for Healthcare Improvement (IHI) Open School are QI-based programs relevant for medical student training of teamwork-related skills [10-12]. IHI modules were originally assigned as the result of an initiative by the College of Medicine to incorporate health systems science topics into the clinical clerkships. These modules provided foundational knowledge on QIt principles. TeamSTEPPS® offers training on team dynamics influencing quality improvement initiatives. Therefore, we hypothesized that after training with the TeamSTEPPS® 2.0 curriculum, medical students would become more adept at identifying areas of improvement within team settings [13,14]. Previous studies have also shown promising results using medical student reflections to better understand the “hidden curriculum,” referring to knowledge obtained experientially as opposed to within a classroom setting [15,16]. Prior student reflection-based work on interprofessional experiences focused on geriatrics and emergency medicine settings, but not pediatric settings [6,17,18]. Notably, the medical students within previously reviewed studies had no defined training in QI before reflecting on their experiences [19].

Medical students are pivotal contributors of input to drive the improvement of teamwork, communication, and leadership in the medical field. When they enter practice, they will be responsible for leading and contributing to healthcare teams related to patient care and/or quality initiatives. To our knowledge, no prior studies have combined QI methodology training with medical student narrative reflections. We further explored this perspective by utilizing a grounded theory approach to process the narrative findings and create a figure illustrating interprofessional team performance. By combining these elements, our study addresses knowledge gaps that currently exist due to the lack of medical student reflection on team-based care, specifically in pediatric settings.

This study was designed to explore what medical students view as significant positive and negative elements of interprofessional care in a pediatric inpatient unit. As participants in care who are not often given a voice in how interprofessional teams function, medical students provide critical insight as learners, contributors, and observers of others on the interprofessional team. This backdrop, paired with QI training in TeamSTEPPS® and IHI Open School, provided the novel setting for our research. The objective of this study was to explore the medical students’ reflections after observing interprofessional team functioning and how those reflections relate to principles of QI and team dynamics.

## Materials and methods

Using a survey card based on TeamSTEPPS®, reflections written by medical students were analyzed using qualitative thematic analysis techniques. This study was classified as not involving human subjects by the University of Nebraska Medical Center Institutional Review Board based on the project being a program improvement initiative. This work was also presented as a poster at the 2018 Institute for Healthcare Improvement annual meeting in Orlando, Florida. 

Sampling strategy

During academic years 2016-2018 at a single U.S. allopathic medical school, 183 of 251 medical students in year 3 (M3s) completed a survey card-based evaluation of an interprofessional team entitled “Performance Assessment of Communication and Teamwork.” All of the medical students participated in required TeamSTEPPS® training and completed IHI Open School QI 101-106 courses (figure in the Appendices) as part of the pediatric clerkship curriculum at the beginning of the clerkship. The survey card asks students to rate team interactions based on TeamSTEPPS® domains of team structure, leadership, situation monitoring, mutual support, and communication. Additionally, reflections based on three different prompts were asked based on module 5 from IHI Open School. Specifically, the prompts focused on communication breakdowns, suggested improvements for team effectiveness, and observations of team members resistant to change.

Students were informed that their survey cards with identifying marks would not be shared with supervisory attendings or program leadership, but they were required to complete one survey card by the end of the clerkship to receive their clerkship grade. The QI-trained students evaluated interprofessional teams in two acute care hospitals: a university-affiliated multidisciplinary hospital and a freestanding children’s hospital. Each student did a rotation of a minimum of three weeks on hospital teams during their pediatric clerkship, and each team included both an attending faculty pediatrician and a resident or fellow supervisor for the student. Many teams also included other members, most frequently advanced practice providers (physician assistant or nurse practitioner), dieticians, and pharmacists, which students noted using a checklist of clinical professional roles in the organization. 

**Figure 1:**
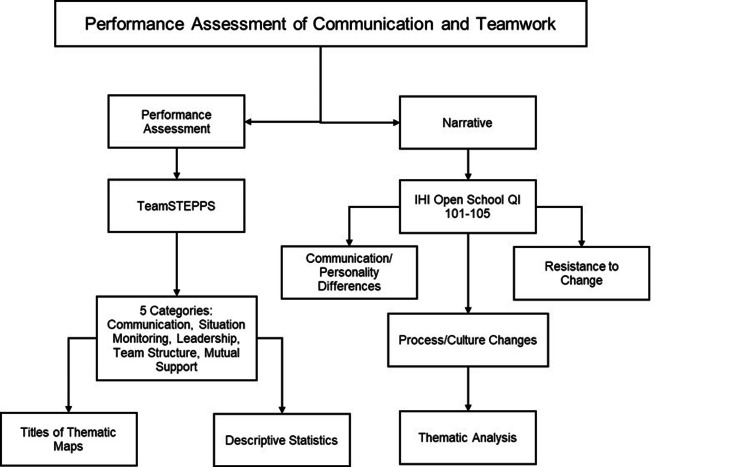
Data analysis process Clerkship students completed an assignment entitled ‘Performance Assessment of Communication and Teamwork’ that had two components. First, an assessment of a clinical team from poor to excellent using the TeamSTEPPS framework. The responses were clustered into the 5 TeamSTEPPS categories, and descriptive statistics and thematic maps were utilized to characterize the teams being assessed by students. The second component was a narrative reflection based upon a writing prompt developed from the objectives of the IHI Open School QI 101-105 coursework. These narratives were used for thematic analysis of team performance in pediatrics. Note: Nurse and Nurse Practitioner were separate categories. Nurses deliver bedside care based upon the orders received from the providers; nurse practitioners are serving in a provider role and generate care plans IHI: Institute for Healthcare Improvement

The M3s evaluated one of the interprofessional pediatric teams they rotated with using the five skill domains of TeamSTEPPS® (situation monitoring, communication, leadership, team structure, mutual support). Average team ratings were calculated on a scale of 1-5 (1=poor to 5=excellent performance). In addition, narrative responses were collected from a selection of three prompts corresponding to the IHI QI 105 module, “Leading Quality Improvement.” The three prompts addressed feedback surrounding communication, culture, and process change recommendations, and resistance to change, respectively. Figure 1 outlines the data analysis approach we used when processing the survey cards.

Thematic analysis and maps

A thematic analysis was conducted of the narrative responses using a six-phase approach: data familiarization, coding, searching for themes, reviewing themes, defining/naming themes, and writing up [20]. The inductive nature of the thematic analysis derived new meanings from the data presented [21,22]. Phase 1 included manual transcription of the 183 handwritten responses. Phase 2 entailed reading each response and identifying key words. Phase 3 involved grouping the words to generate themes. Phase 4 entailed refining, combining, and renaming the generated themes (i.e., combining “Stress” and “Burnout”). Phase 5 involved naming 23 primary themes and 17 subthemes. Phase 6 involved creating a comprehensive table including the themes and subthemes. Narrative data obtained from the reflections were synthesized into thematic maps by grouping each theme under the applicable TeamSTEPPS® skill domains.

Valence rating system

A valence rating system was developed to quantify the narrative responses as overall positive/attractive or negative/aversive to increase reflexivity [23]. The valence rating system assigned values on a scale of 1-7 influenced by the existing Empirical Valence Scale (EVS), with 1 being high negative valence (all the comments were negatively valenced) to 7 being high positive valence (all the comments were positively valenced) [24]. Scores of 1-3 were negatively valenced, a score of four was neutral, and scores of 5-7 were positively valenced.

Qualitative analysis process

The thematic analysis and assignment of valence ratings were independently conducted by two investigators (CN, RB) who individually read each student reflection and employed inductive coding to identify themes. Inter-rater reliability for the valence rating system was measured, and the Cronbach’s alpha was 0.958, indicating a high internal consistency. Subsequently, additional narrative checking and valence assurance were conducted by a third investigator (KS) to randomly review and verify the coding scheme. Finally, discussion and consolidation of the most frequently observed topics were conducted by the co-authors (KS, CN, RB).

Interprofessional team performance conceptual model

Using the TeamSTEPPS® and valence ratings, the 10 highest-scored and 10 lowest-scored survey cards were isolated and evaluated. The highest-scored cards received a TeamSTEPPS® score of at least 4.5/5.0 or an average valence rating of at least 6.0/7.0. The cards meeting both criteria were labeled as the “10 Highest Scored Cards,” having an average TeamSTEPPS® score of 4.9/5.0 and an average valence rating of 6.5/7.0. Similarly, the lowest-scored cards each received a TeamSTEPPS® score of at most 3.6/5.0 or an average valence rating of at most 2.0/7.0. The cards meeting both criteria were labeled as the “10 Lowest Scored Cards,” having an average TeamSTEPPS® score of 3.5/5.0 and an average valence rating of 1.6/7.0.

In this study, interprofessional team performance was evaluated using medical students' ratings and perceptions after they received the QI-based training. After conducting the thematic analysis, the emergent themes consistently displayed by teams with the highest and lowest performance ratings were further examined using a grounded theory approach [25,26]. The medical students’ explanations within the 10 Highest Scored and 10 Lowest Scored survey cards provided the foundation for the development of our conceptual model, an evidence-based graphic that displays elements of both high and low-performing interprofessional teams. We tied the four key attributes of interprofessional team performance to one or more of the TeamSTEPPS® Key Principles.

## Results

Demographic data

A summary of demographic data is presented in Table 1.

**Table 1:** Results of “Performance Assessment of Communication and Teamwork Survey” (n=183) Two students wrote reflections from a team that was led by a non-physician (nurse practitioner or physician assistant). Therefore, 181 teams had a physician on the team, but medical students returned 183 total cards SD: standard deviation

Survey results			
Professions represented in the team (>1 choice allowed)			
		N	%
Medicine		181	99.40%
Nursing		163	89.60%
Pharmacist		135	74.20%
Physician's assistant		47	25.80%
Other		101	55.50%
Nurse practitioner		40	21.90%
Social work		27	14.80%
Dietician/nutritionist		52	28.40%
Completed survey with other health profession students (>1 choice allowed)			
Medicine		62	34.10%
Nursing		8	4.30%
Pharmacy		17	9.30%
Other		1	0.01%
Setting of responses (>1 choice allowed)			
Pediatric ICU		17	9.30%
Neonatal ICU		40	22%
Hematology-oncology		20	11%
Med-Surg inpatient		98	53.80%
Other		23	12.60%
TeamSTEPPS skill domains (team performance low-1 to high-5)			
	Mean	Median	SD
Team structure	4.48	5	0.56
Leadership	4.46	5	0.64
Situation monitoring	4.28	4	0.77
Mutual support	4.54	4	0.58
Communication	4.29	4	0.74

Thematic analysis and maps

**Figure 2:**
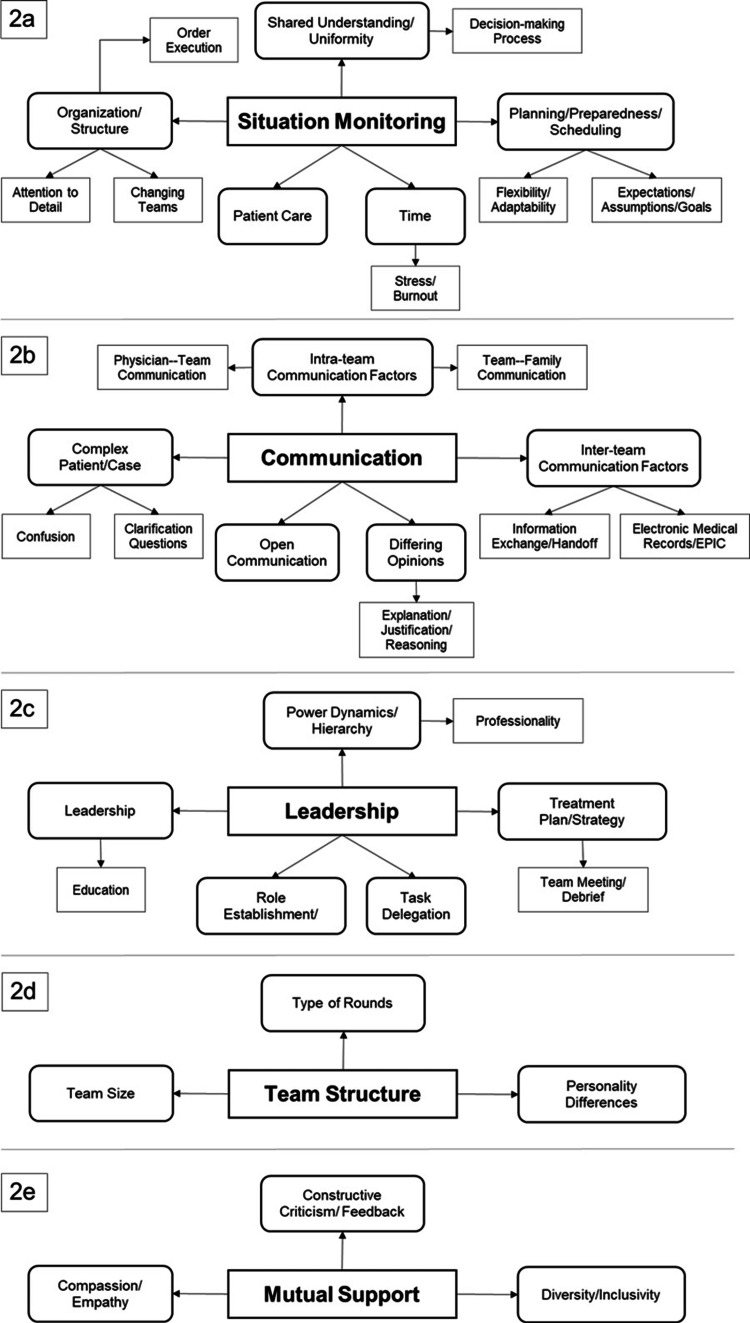
Thematic maps Based on the inductive thematic analysis of reflections, thematic maps were developed for the five TeamSTEPPS categories, including subthemes supporting the categories. The maps are: 2a - situation monitoring; 2b - communication; 2c - leadership, 2d - team structure, and 2e - mutual support

The inductive thematic analysis of the 183 narrative reflections generated 40 themes, which were subsequently grouped under the five TeamSTEPPS categories based on their representativeness of each category. Students preferentially reflected upon opportunities to improve team performance, with mentions per TeamSTEPPS® skill domain inversely correlating with domain-scaled rating (i.e., situation monitoring had the most mentions at 250 but the lowest overall rating at 4.28). The primary themes in each category were identified (23 total; up to five in each category), and the remaining 17 themes were grouped under the top themes and labeled “subthemes.” A thematic map was generated for each TeamSTEPPS® category (Figures 2a-2e). Of note, two themes, process change and culture change, were not included in the thematic maps since these themes applied across all TeamSTEPPS® categories. The three themes with the greatest saturation were categorized under patient care (67 mentions, 37% of students), type of rounds (66 mentions, 36% of students), and intra-team communication factors (50 mentions, 27% of students).

Patient Care

The phrase “patient care” was not directly mentioned on the student survey card, yet it was the most commonly mentioned theme in the narrative data. This is perhaps the most insightful preliminary finding of the study, as it indicates students’ desire to deliver effective, thoughtful patient care. One student observed an instance of resistance to change related to patient care:

I did witness times when members of teams that I worked with resisted change in procedure or patient care. The majority of the reasons behind the resistance were the increased level of burden on that individual. I will say that I saw this more with team members towards the top of the chain of command and less at the bottom. The resistance centered around more work, an increased amount of time required, or new procedures/steps to complete. This is, however, where I saw the best teamwork. In these situations, the team would work together and divide tasks appropriately, so that the care for the patient was optimized. At the end of this experience, I realized that the ultimate goal of the care team was the outcome for the patient (narrative 133).

This student presented two examples of team functioning. The observation suggests that teams should focus less on the hierarchical roles of the individual members of the team to ensure the highest quality of patient care. 

Type of Rounds

The family is often more involved during pediatric rounds, especially when the patient is too young to communicate independently [27]. Students frequently commented on the structure and content of daily patient rounds. The two types of rounds mentioned were family-centered bedside rounds and table rounds. Regardless of the rounding format, there was a consensus that the family should be included in patient care decision-making. The current literature suggests that family-centered bedside rounds can help improve communication, decision-making, and learning in pediatric care [28,29]. One student compared patient-centered bedside rounds with table rounds at two separate hospitals:

When seeing the difference in how (hospital 1) inpatient and (hospital 2) conducts rounds, I believe that patient-centered rounds greatly improve effectiveness. It often seemed that different members of the team got different stories from family members, which made care difficult. At (hospital 2), having the family deeply involved with the care empowered the patient and family to let all members of the team know what they wanted and how the patient was doing. This eliminated barriers when the team later constructed a plan of care. I think a patient-centered rounds approach also changes the culture so that everyone views the decisions they make related to patient care as a personal and very important act. It helps one imagine what one would want done if it were your family member. In conclusion, I thought it was very interesting to see two very different approaches to conducting rounds. While both have their pluses, I think any time you can engage the whole team with a patient, the patient benefits (narrative 59).

Students also proposed that teams benefit when both pre-rounding and a debriefing session occur after rounds:

I think all those in leadership roles at rounds have promoted an atmosphere of understanding, and the particular day at rounds that I am writing about demonstrated excellent communication, role identification, and responsibility distribution. Debriefing sessions following rounds to identify lapses are particularly impressive and useful (narrative 107).

As the student above observed, debriefing sessions help identify lapses in communication and make sure every team member has a clear understanding. Furthermore, the following student suggested having a discussion or meeting before rounds to keep every member of the team involved and updated:

They have a meeting with the entire healthcare team before the patient visit, which not only enhances patient care but also ensures that the physician is fully addressing the family's needs associated with each patient (narrative 154).

The student reflections recommended prioritizing family-centered bedside rounds, pre-rounding, and having a debriefing session as key recommendations centered around improving care delivery.

Process Change and Culture Change

Organizational change has been described by Lewin's 3-stage model as an intentional process that 1) unfreezes, 2) changes, and 3) re-freezes unwanted “frozen” behaviors to new, more desirable behaviors [30-32]. The IHI Open School defines two change categories: process change and culture change. Process changes involve a change in the method of task performance, and culture changes involve transforming values and perspectives [33].

Within the narratives, students offered recommendations for both process and culture changes. The process change recommendations were centered around information exchange, and the culture change recommendations were centered around inclusivity. This student proposed a change that addressed both information exchange and inclusivity:

A possible improvement would be to add a quality improvement question to the end of a presentation that is open to the team. If someone has an idea, they can say it then. Adding this would be both a process and culture change. It would add a step to rounds that would take time, but it would change the culture to try to be more inclusive of all professions during rounds, while also emphasizing the importance of constantly improving patient care (narrative 28).

Another student focused solely on information exchange, recommending that only a few members of the team convey decisions to the family:

The culture change is one of increased sensitivity to the fears of the patient, away from one of maximum efficiency. Similarly, the process change is made to ensure the families are not overwhelmed with the number of people talking to them during a sensitive time in their lives. These changes are important to provide compassionate care because they ensure that the patient and their family are appropriately informed while minimizing the level of discomfort during discourse with the care team (narrative 61).

Furthermore, this next narrative involved a process change recommendation:

It's best to pre-plan how the encounter will go together before entering the patient's room. I think this process change would result in more effective communication with the patient, and it would allow for a more patient-centered interaction vs. a physician-centered interaction (narrative 5).

All three of these narrative examples provided recommendations on improving patient care through either team inclusivity or greater inclusion of the family in decision-making.

Most and least favorable cards

After examining the 10 highest- and lowest-scored survey cards, we discovered consistent thematic markers inherent to both high and low-performing interprofessional teams supported by prior studies [33-35].

Most Favorable Cards

High-performing teams cultivated open communication, had a strong sense of shared understanding among team members, established clearly defined team roles, and displayed an organized team structure.

Open communication in pediatric settings is important for continuous information sharing, preventing miscommunications between team members, and establishing a sense of trust and respect [36]. One student commented:

We were all free to voice our thoughts, including students. Everyone respected each other, which developed that trusting environment. I honestly didn't notice any communication barriers because of the reasons outlined above (narrative 24).

Having a shared understanding among team members is another important element of a high-functioning pediatric medical team, as evidenced by this student’s narrative:

I felt that this team did an excellent job overall of understanding the role of each member of the interprofessional team. The physicians and residents also respected that the nurses, in general, spent more time with the patients and sometimes had a better understanding of how the patient may respond, due to previous trials (narrative 23).

By having a clear understanding of each team member’s roles, interprofessional teams establish a shared mental model that improves teamwork and enhances patient care [37]. One student observed:

The roles for each team member are clear and well-defined. Each team member worked within their respective role, executing their jobs fully so that the other team members are able to complete their work promptly (narrative 57).

Within multiple reflections, students emphasized organization and structure as two other important aspects of teamwork in pediatric settings, particularly in complex inpatient environments:

I was amazed by the constant communication and execution of orders by all staff. I would watch the residents put in orders and immediately see nursing staff executing them. I also witnessed the nursing staff ask the residents and attendings to put in orders so they could assist the patients, which they did immediately (narrative 58).

Least Favorable Cards

Our results showed teams with the lowest scores demonstrated unhealthy power dynamics, lacked role clarity, displayed ineffective information exchange, and struggled with task delegation.

Pre-existing power perceptions among team members cause team communication, cooperation, and mutual respect to suffer [38]. This reflection described unhealthy power dynamics:

There were numerous times when nursing staff were designated with discharge planning or the plan to progress care. Their concerns were heard by the attending, but occasionally he did not allow them to continue explaining their reason, especially when they continued to defend their point of view despite the attending's explanation against what they were saying (narrative 90).

Another student discussed an instance of role ambiguity due to inconsistencies between physician team leaders, offering that simply establishing a leader is not sufficient for high-performing teamwork, especially in the case of continually changing teams:

There were times when it was unclear what roles/expectations were. The physician was clearly the leader of the group, but because different physicians rotated in and out of the inpatient service every week, expectations changed week to week, and it was on the other members of the group to figure out what the new roles/expectations were going to be (narrative 91).

Medical students often shared examples of ineffective information exchange between providers, which would hinder the team’s interprofessional functioning:

There were times each provider would bombard the patient with questions. Although they took turns speaking, it got to be a little overwhelming, and information would get lost, resulting in re-asking the same question (narrative 29).

The barrage of questioning by different providers may inadvertently leave others on the team unclear about what to do next. These types of encounters might benefit from a debriefing after seeing the patient to ensure other members of the team are clear on the priorities and next steps in the patient’s care.

Lastly, this student responded to a breakdown in task delegation, which resulted in the delivery of suboptimal patient care:

This was a very simple lack of communication that, unfortunately, was not addressed immediately…everyone on the team expected someone else to handle it, and no one did. This problem could have been solved by someone stepping up and claiming the task or delegating the task (narrative 43).

Conceptual model for interprofessional team performance

**Figure 3:**
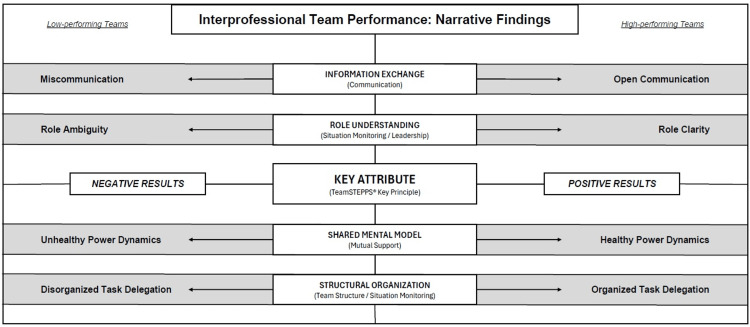
Narrative findings on interprofessional team performance Student narratives were scored as having a positive or negative valence. The inductive findings from the highest and lowest scored cards were analyzed using a grounded theory approach and the themes organized around the four key attributes of interprofessional team performance

The inductive findings from the highest- and lowest-scored cards were analyzed using a grounded theory approach [25], which resulted in the development of “Interprofessional Team Performance: Narrative Findings” (Figure 3). As noted, the four most common themes within the 10 highest-scored survey cards were role establishment, open communication, shared understanding, and organized structure. The top four themes within the 10 lowest-scored survey cards included role understanding, information exchange, power dynamics, and task delegation.

We found that the top four themes within the highest- and lowest-scored survey cards functioned as opposite ends of four key attributes within interprofessional team performance in pediatric settings (information exchange, role understanding, shared mental model, structural organization). Each attribute also shared characteristics with one or more of the five TeamSTEPPS® Key Principles. In support of these findings, the students’ narratives identified:

- Lack of* information exchange* was strongly correlated with breakdowns in communication

- An inclusive culture of *open communication* led to an improvement in the exchange of information

- Effective team leaders clearly outlined team members' roles, resulting in *role clarity*

- Ineffective team leaders did not articulate roles or change expectations for team members, resulting in *role ambiguity*

- Teams with *unhealthy power dynamics* lacked understanding of each team member’s expected contributions

- Teams with *healthy power dynamics* demonstrated a *shared mental model *and mutual support for all team members, with an understanding of their expected roles and contributions

- Effective situation monitoring by *organized *teams resulted in *efficient task delegation*

- Disorganized teams demonstrated *inefficient task delegation* and reduced situational awareness

Our evidence-based cohesive figure provides opportunities to inform future studies on the importance of each of the facilitators and barriers surrounding team performance. Team member feedback on these themes could also be sought to improve interprofessional team performance in pediatric settings, both educationally and experientially.

## Discussion

Our study explored the drivers of and barriers to effective interprofessional team performance in pediatric settings using medical student reflections in the context of QI-based training. Receiving training in TeamSTEPPS® and IHI Open School in advance of completing the survey cards provided students with the awareness to articulate their observations on potential opportunities for improvement in patient care. The inductive results of the thematic analysis enabled the creation of a cohesive figure centered around four key attributes of interprofessional team performance that have promising implications for bridging QI and medical education (Figure 3). This figure can be used in future research as an applied framework to develop QI interventions to improve interprofessional team-based care.

Effective team performance requires coordination through the modification of team behaviors (communication, cooperation, leadership, monitoring) [33]. After completion of the training, the participating students in our study gained a baseline of knowledge regarding communication, teamwork, and quality improvement. This resulting knowledge allowed them to effectively comment on themes pertaining to interprofessional team functioning. The analytic approach we took when processing the survey cards (Figure 1) allowed us to process the data in a manner that led to the subsequent creation of Figure 3. The finding that students most frequently selected to reflect upon opportunities for improvement in team performance highlights the potential for focused quality improvement training to uncover the “hidden curriculum” and to create opportunities for students to generate insights into understanding high and low-performing healthcare teams.

High-performing teams display a culture where team members feel empowered to speak up about potential concerns [39,40]. We found that open communication empowered each team member to freely share thoughts and information. This increased information exchange between team members and the patient/family. Effective information exchange gives team members access to understandable and clear information to guide patient care [27]. The teams with high performance ratings demonstrated a culture of open communication among members, whereas teams with low ratings displayed a lack of effective information exchange, leading to miscommunications. Therefore, our study supported addressing the communication culture as a way to help improve the team’s overall capacity to deliver exemplary patient care.

The most productive teams are those that balance professional autonomy, effective delegation, and interdependence [41]. Our research expanded upon this notion by demonstrating that an understanding of roles and responsibilities is necessary for successful interprofessional teams. Students noticed the roles of each team member were established through a combination of leader-directed information, background experience, and collaboration. The results suggested that team members worked effectively within their own areas of expertise when roles were clear, but ambiguity resulted when a lack of guidance was given from the team leader. The narratives ultimately offered that effective leadership promotes clear role establishment and understanding.

Medical teams are multifaceted in nature, and complex power dynamics are often present between faculty, other healthcare professionals, and trainees [38]. The student reflections revealed that when teams displayed mutual support for all team members, clarity about individual responsibilities was identified, and team performance was enhanced. However, when teams failed to achieve a balance of power and support, the results were unhealthy power dynamics and team members feeling unsupported. Medical student reflections suggested that teams with high performance ratings appeared to deliver more efficient care with greater accountability and organization per the medical student reflections. These teams exhibited more clarity of responsibility and shared accountability for the completion of daily tasks. In future applications of this research, focusing efforts on structural organization has the potential to improve patient care delivery, efficiency, and outcomes.

As previously mentioned, the figure developed from the results of this study can serve as a basis for identifying teamwork facilitators and barriers to improve interprofessional team performance in pediatric medical settings. Using the findings of this study, we propose that promoting open communication, role clarity, shared understanding, and organized task delegation will improve patient care outcomes. We also propose that directing efforts to improve information exchange, role understanding, power dynamics, and task delegation will result in positive outcomes for patient care. For future applications, our graphic can be implemented within many facets of team-based care, from medical education to interprofessional culture.

Our study addresses existing knowledge gaps surrounding the medical student perspective on interprofessional pediatric team-based care. After analyzing narrative data from student participants on the clinical team who have received prior QI-related training, we were able to identify key attributes that students emphasize as being beneficial or detrimental to team performance and patient care. Past studies have examined medical student narrative reflections within experiential learning [15,16,19,42]. Analysis of student reflections suggested that this concept of having medical students use QI-relevant knowledge to actively evaluate team performance may lead to improving team performance. The evidence-based figure we developed can further identify interprofessional teamwork opportunities within pediatric medicine. 

This study has a few limitations. Our study involved a single allopathic medical school reporting M3 experiences in pediatric clerkship settings, and hence, the results may not be generalizable to other medical schools or other clerkship settings. For example, the pediatric setting may differ from experiences in adult care settings, particularly with respect to communication needs and style of rounds. Of note, this study was conducted before the COVID-19 pandemic, and as such, results may not be generalizable to the current state of healthcare. Follow-up studies using narrative data from medical students in the post-COVID-19 era could be conducted to investigate if results evolved during and after the pandemic. Future efforts should also include applying the framework we have constructed to studies involving students from other medical schools and sampling other interprofessional team members to broaden and compare different perspectives, as well as including interprofessional team members on the study team. Additionally, this model may be implemented across clerkships or longitudinally to explore team dynamics in different contexts by using end-of-clerkship evaluations.

## Conclusions

This study aimed to expand upon current knowledge related to interprofessional team performance by encouraging medical students to reflect on opportunities to enhance communication and teamwork after receiving training in QI methodology. Student narratives were analyzed to identify team performance elements that future physicians deem most influential regarding interprofessional care. Through this opportunity to reflect upon interprofessional team performance, students gained a deeper understanding of the dynamics that influence care delivery and quality. By fostering this awareness and valuing student perspectives, interprofessional medical teams can become more responsive and better equipped to support both high-quality patient care and meaningful student learning experiences.

## Figures and Tables

**Figure 4:**
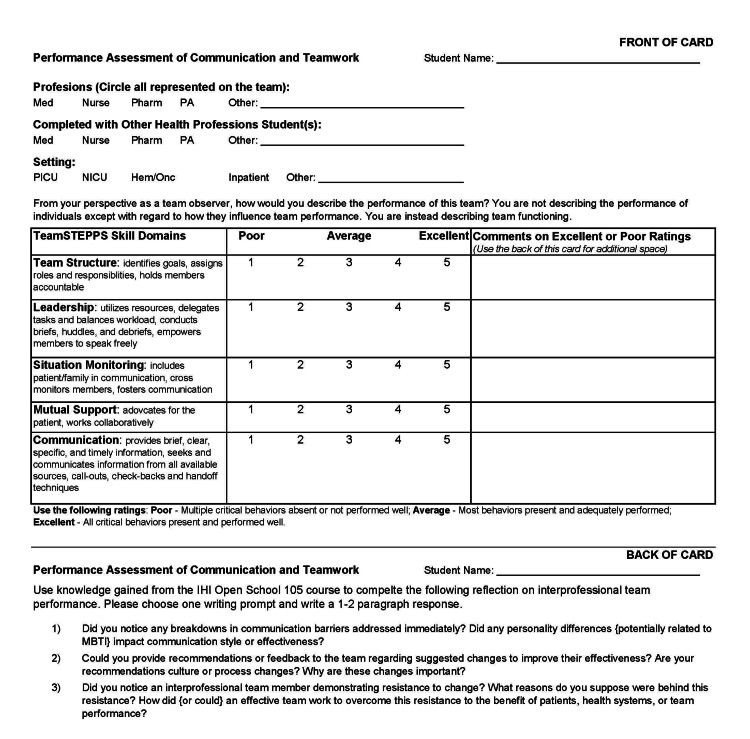
“Performance Assessment of Communication and Teamwork” card Students completed this card and responded to the prompts on the reverse side. The front and back of the card are shown in the figure
